# Metformin and gefitinib cooperate to inhibit bladder cancer growth via both AMPK and EGFR pathways joining at Akt and Erk

**DOI:** 10.1038/srep28611

**Published:** 2016-06-23

**Authors:** Mei Peng, Yanjun Huang, Ting Tao, Cai-Yun Peng, Qiongli Su, Wanjun Xu, Kwame Oteng Darko, Xiaojun Tao, Xiaoping Yang

**Affiliations:** 1 Department of Pharmacy, School of Medicine, Hunan Normal University, Changsha, Hunan 410013, P. R. China; 2 TCM and Ethnomedicine Innovation & Development Laboratory, Hunan University of Chinese Medicine, Changsha, Hunan 410208, P. R. China

## Abstract

EGFR is a potential therapeutic target for treating bladder cancer, but has not been approved for clinical use yet. Metformin is a widely used antidiabetic drug and has demonstrated interesting anticancer effects on various cancer models, alone or in combination with chemotherapeutic drugs. The efficacy of gefitinib, a well-known EGFR tyrosine kinase inhibitor, combined with metformin was assessed on bladder cancer and underlying mechanisms were explored. This drug combination induced a strong anti-proliferative and anti-colony forming effect and apoptosis in bladder cancer cell lines. Gefitinib suppressed EGFR signaling and inhibited phosphorylation of ERK and Akt. Metformin amplified this inhibitory effect and enhanced gefitinib-induced activation of AMPK signaling pathway. *In vivo* intravesical treatment of metformin and gefitinib on syngeneic orthotopic mice confirmed the significant inhibitory effect on bladder tumor growth. These two drugs may be an excellent combination for the treatment of bladder cancer through intravesical instillation.

Bladder cancer is one of the most common cancers of the urinary tract in the world, accounting for about 74,000 new cases and 16,000 deaths in the United State in 2015^1^. Although radical cystectomy with chemotherapy has been applied for treating bladder cancer patients as the standard clinical administration and exerted efficient benefits, recurrence and metastasis take place frequently^2^. To prevent recurrence and progression, intravesical chemotherapy or immunosuppressive agents have been widely used after radical cystectomy^3^,^4^. However, these supplemental methods are largely restricted with various degrees of side effects such as bone marrow suppression, allergic reactions and etc^5^. Thus, there is an unmet demand to develop safe and effective administration strategy for treating bladder cancer.

Targeted therapy directed at specific molecular pathways is a promising avenue. A number of large-scale molecular studies have been conducted in bladder cancer^6^,^7^,^8^ and highlighted several potential therapeutic targets such as epidermal growth factor receptor (EGFR)^9^. EGFR pathway plays a critical role in cell proliferation, apoptosis, differentiation, migration and angiogenesis^10^,^11^,^12^ and the expression of EGFR/ERBB2 correlates with higher tumor grade/stage and poorer prognosis in bladder cancer^13^,^14^,^15^,^16^. It has been shown that EGFR-TKI such as gefitinib is helpful for the adjuvant treatment of primary bladder cancer, however, the clinical trials suggested that it exerted modest efficacy in patients with previously treated metastasis UC(SWOG 0031)^17^. This may be explained by the biodiversity of the molecular signaling pathways implicated in bladder cancer pathogenesis. In general, targeting one single specific cellular pathway results in up-regulation of other pathways. Thus, targeting multiple molecular pathways may be a better option for treating cancer including bladder cancer.

Warburg effect has been proposed as one typical characteristic of tumors marked by abnormality of metabolism. There is renewed interest in developing novel anti-cancer breakthroughs modulating metabolism to limit neoplastic growth. Reversing Warburg effect might be a profound strategy for cancer treatment and is becoming a hot research area. Surprisingly, recent report has demonstrated that inhibition of EGFR signaling pathway is able to reverse this effect^18^.

At the other hand, metformin, a widely prescribed drug for treating type II diabetes, is one of the most extensively recognized metabolism modulators. It showed inhibitory effect in various cancer cell lines and xenograft cancer models and sensitized chemotherapy drugs^19^. Therefore, we hypothesize that combination of EGFR-TKI and metformin exert synergistic effect for killing bladder tumor.

In another report, we described that metformin showed significant inhibitory effect on bladder tumor growth in syngeneic orthotopic model through intravesical administration^20^. In this study, we aim to examine the effects of a combined treatment of metformin with gefitinib, a selective EGFR-TKI in bladder cancer and explore their mechanisms underlying.

## Results

### The effects of gefitinib, alone or combined with metformin on bladder cancer cell proliferation

**Figure 1:**
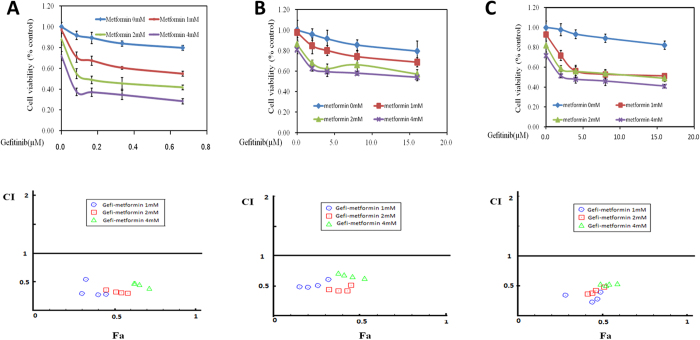
Treatment with gefitinib alone or combined with metformin on cell proliferation of 3 bladder cancer cell lines. (**A**) Gefitinib combined with metformin inhibited MB49 proliferation synergistically. Above: Cell viability was assessed with 48 hour gefitinib at 0, 82.5, 165, 330, 660 nM alone or gefitinib combined with various concentrations (1, 2, 4 mM) of metformin treatment. Below: Combination index (CI) among the combinations of two drugs were calculated using CompuSyn software. If CI = 1, it denotes additivity; if CI > 1, it denotes antagonism; if CI < 1, it denotes synergism. CI values in the vast majority of combinations were less than 0.5, indicating moderately strong synergism. (**B,C**) Gefitinib combined with metformin inhibited T24 and UMUC3 proliferation synergistically. Above: Cell viability was assessed with 48 hour gefitinib at 0, 2, 4, 8, 16 μM alone or gefitinib combined with various concentrations (1, 2, 4 mM) of metformin treatment in T24 and UMUC3, respectively. Below: Combination index (CI) among the combinations of two drugs were calculated using CompuSyn software. If CI = 1, it denotes additivity; if CI > 1, it denotes antagonism; if CI < 1, it denotes synergism. Results are presented as the median of 5 independent experiments.

To evaluate the anti-proliferative effects of different doses of gefitinib, alone and in combination with metformin, we performed a MTT assay on bladder cancer cell lines MB49, T24 and UMUC3. As shown in Table 1, IC50 values for MB49, T24 and UMUC3 are 0.75 μM, 25.74 μM, 25.90 μM respectively. Generally, at the range of tested concentrations, gefitinib alone didn’t show dramatic inhibitory effects. Adding metformin exhibited a profound synergistic effect as assessed by CompuSyn software (Fig. 1).

### Suppression of colony formation

**Figure 2:**
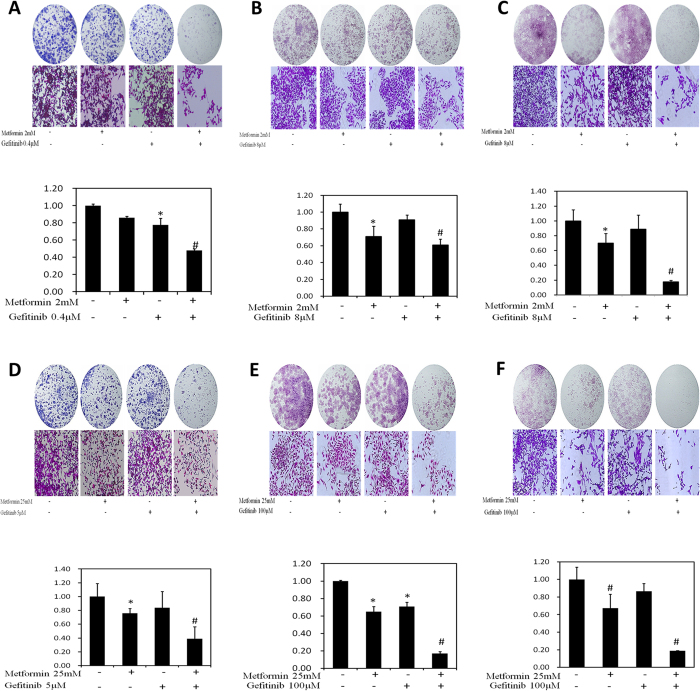
Evaluation of colony suppression of Gefitinib alone or combined with metformin on 3 bladder cancer cell lines. (**A–C**) Clonogenic assay was assessed after 7 day Gefitinib treatment alone or combined with metformin and wells were stained with crystal violet at the end of the experiment. (**A**) Clonogenic assay in MB49 was conducted with the treatment of 2 mM metformin, 0.4 μM Gefitinib and their combination. Above: The full view of wells were taken through stereomicroscope and images were taken through an inverted microscope with ×10 magnification. Below: the quantification of colony was determined by microplate area scan at OD 550 nm, Results are presented as the median of 5 independent experiments (*P < 0.05, ^#^P < 0.01 vs control). (**B,C**) Clonogenic assay was conducted with the treatment of 2 mM metformin, 8 μM Gefitinib and their combination in T24 and UMUC3, respectively. The full view of wells and their quantification were obtained through the same method as described in MB49. Results are presented as the median of 5 independent experiments (*P < 0.05, ^#^P < 0.01 vs control). (**D–F**) Colony formation assay was carried out with two hour treatment at labeled concentrations, twice per week for two weeks and stained with crystal violet at the end of the experiment. (**D**) Clonogenic assay in MB49 was conducted with the treatment of 25 mM metformin, 5 μM Gefitinib and their combination. The full view of wells and their quantification were obtained through the same method as described in MB49. Results are presented as the median of 5 independent experiments (*P < 0.05, ^#^P < 0.01 vs control). (**E**,**F**). Clonogenic assay was conducted with the treatment of 25 mM metformin, 100 μM Gefitinib and their combination in T24 and UMUC3, respectively. The full view of wells and their quantification were obtained through the same method as described in MB49. Results are presented as the median of 5 independent experiments (*P < 0.05, ^#^P < 0.01 vs control).

We next examined colony formation in the presence of gefitinib alone or combined with metformin. In regular continuous fashion, we found that gefitinib alone at concentration between 0 to 0.4 μM in MB49, 0 to 8 μM in T24 and UMUC3 exhibited little inhibitory effect on bladder cancer cells. However, this inhibitory effect increased when combined gefitinib with 2 mM metformin (Fig. 2A–C).

To mimic intravesical treatment, we designed an intermittent treatment protocol with 2 hours drug incubation twice per week for two weeks. Understandably, compared with that in continuous fashion in general, much higher concentrations of these drugs are needed for inhibiting the colony formation, As shown in Fig. 2D,E, the combinations of gefitinib with metformin significantly reduced colony formation in these three bladder cancer cell lines at the indicated concentration.

Taken together, these results have demonstrated the combined use of gefitinib and metformin synergistically inhibits proliferation and colony formation of bladder cancer cells.

### Effects on apoptosis by combined treatment of metformin and Gefitinib

**Figure 3:**
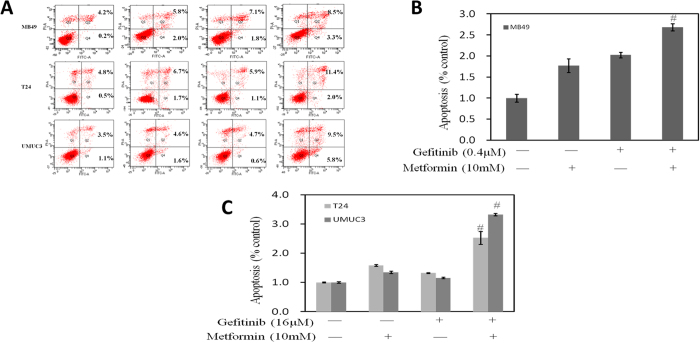
Evaluation of apoptosis of gefitinib alone or combined with metformin on 3 bladder cancer cell lines MB49, UMUC3 and T24. (**A**) Representative flow cytometry scatter plots of propidium iodide (PI) (Y axis) vs Annexin-fluorescein isothiocyanate (FITC) (X axis). (**B**) Bar charts show quantitative data of average of 3 independent flow cytometry experiments in MB49 cells (*P < 0.05, ^#^P < 0.01 compared with control). (**C**) Bar charts show quantitative data of average of 3 independent flow cytometry experiments in T24 and UMUC3 cells (*P < 0.05, ^#^P < 0.01 compared with control).

We further asked whether the increased anti-proliferative effect induced by metformin alone and in combination with gefitinib would be the result of an increased apoptosis. Therefore, we analyzed the induction of apoptosis in MB49, T24 and UMUC3 cell lines after 24-hour treatment with metformin as single agent or in combination with gefitinib. As shown in Fig. 3, flow cytometric analysis of UMUC3 cells revealed that few gefitinib-treated cells underwent apoptosis. In contrast, combined treatment with both gefitinib and metformin significantly enhanced the apoptotic cells. Similar trends were observed in T24 and MB49 cells.

### Amplification of metformin on inhibitory effect of gefitinib through inducing AMPK activation

**Figure 4:**
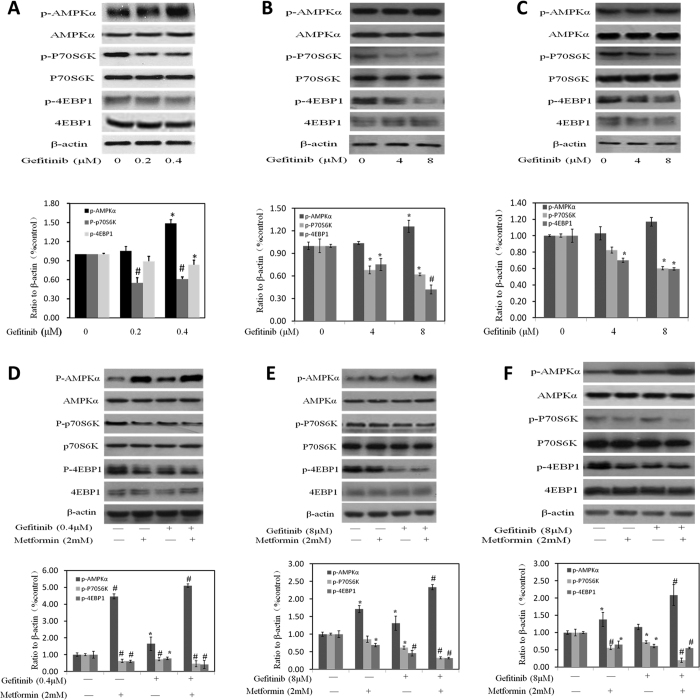
Effects of Gefitinib alone or the combination of Gefitinib with metformin on AMPK intracellular signaling pathways in 3 bladder cancer cell line. (**A–C**) Rrepresent western blottings of p-AMPK, p-P70S6K, p-4EBP1, t-AMPK, t-P70S6K and t-4EBP1 with the treatment of Gefitinib alone. β-actin was included as a loading control. The ratio of different proteins to β-actin was calculated by the band density of Western blots using Image J software. Results are presented as the median of 3 independent experiments (*P < 0.05, ^#^P < 0.01 vs control). (**D–F**) Metformin amplified the effect of Gefitinib on AMPK signaling pathway. (**D–F**) Rrepresent western blottings of p-AMPK, p-P70S6K, p-4EBP1, t-AMPK, t-P70S6K and t-4EBP1 with the treatment of metformin, Gefitinib alone and their combination. β-actin was included as a loading control. The ratio of different proteins to β-actin was calculated by the band density of Western blots using Image J software. Results are presented as the median of 3 independent experiments (*P < 0.05, ^#^P < 0.01 vs control).

AMPK, as an energy sensor, plays an important role in cancer cell metabolism. Another report has shown that metformin significantly activated AMPK signaling pathway in the bladder cancer cell line. To determine whether the activation of AMPK signaling pathways by metformin helped in the inhibitory effect of gefitinib, we investigated the changes of intracellular AMPK signaling pathway after the treatment of gefitinib alone or combined with metformin (Fig. 4). Western blot analysis demonstrated that gefitinib alone activated AMPK and inhibited its downstream signaling proteins such as p-P70S6K or p-4EBP1 in part (Fig. 4A–C). After combining with metformin, a more pronounced decrease of the levels of protein phosphorylation of P70S6K and 4EBP1 was seen (Fig. 4D–F). Three cell lines including MB49, UMUC3 and T24 exhibited similar fashions responding to these treatments.

### Effects of gefitinib treatment alone or combined with metformin on EGFR signaling pathway

**Figure 5:**
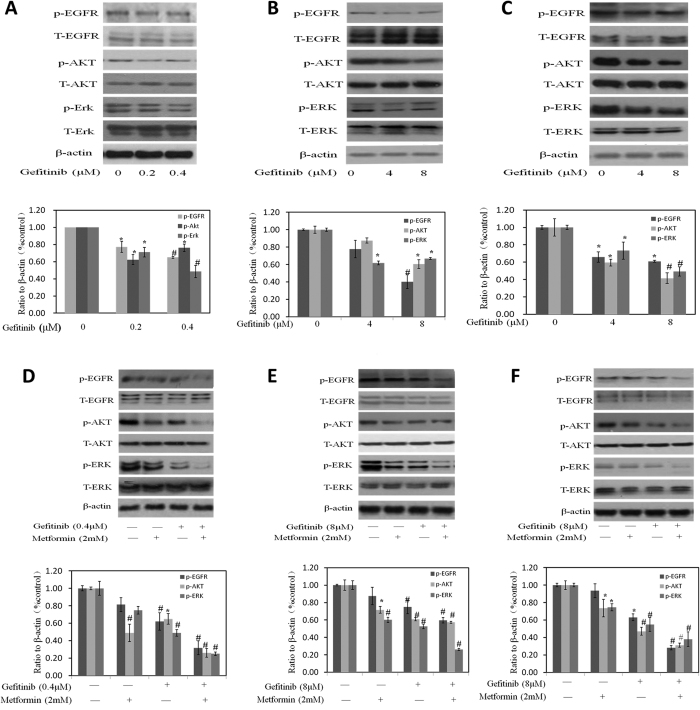
Effects of Gefitinib alone or combined with metformin on EGFR signaling pathway in bladder cancer cells. (**A–C**) Western blotting of p-EGFR, p-AKT, p-ERK, t-EGFR, t-AKT and t-ERK with the treatment of gefitinib alone. β-actin was included as a loading control. The ratio of different proteins to β-actin was calculated by the band density of Western blots using Image J software. Results are presented as the median of 3 independent experiments (*P < 0.05, ^#^P < 0.01 vs control). (**D–F**) Representative western blotting of p-EGFR, p-AKT, p-ERK, t-EGFR, t-AKT and t-ERK with the treatment metformin, gefitinib and their combination. β-actin was included as a loading control. The ratio of different proteins to β-actin was calculated by the band density of Western blots using Image J software. Results are presented as the median of 3 independent experiments (*P < 0.05, ^#^P < 0.01 vs control).

Gefitinib, a selective EGFR tyrosine kinase inhibitor, down-regulated the phosphorylation of EGFR, AKT and Erk while it exerted little effect on total protein of EGFR, AKT and Erk in MB49 (Fig. 5A–C). Another study from our group^20^ proved that metformin inhibited bladder cancer cell growth associated with the decrease of phosphorylated AKT and ERK which are key intracellular mediators of cell survival and proliferation signals. These data together suggest that the synergistic inhibition of metformin and gefitinib on bladder cancer cells may be dependent on both AMPK and EGFR pathways jointing at Akt and Erk. As we expected, metformin caused a slight decrease on EGFR phosphorylation while further decrease was observed after the combination of metformin with gefitinib (Fig. 5D–F). Either metformin or gefitinib decreased the levels of phosphorylation of AKT and ERK and these decreases were strongly expanded while combining metformin with gefitinib (Fig. 5D–F).

**Figure 6:**
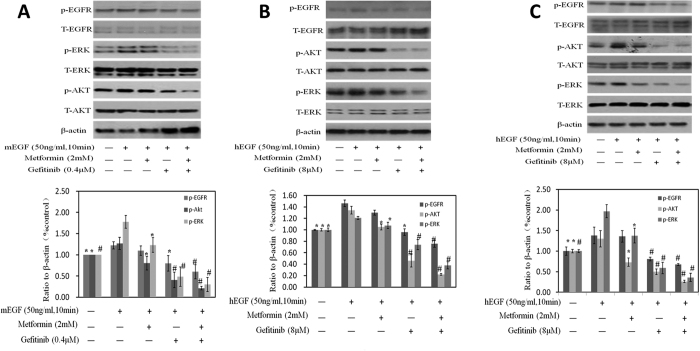
Effects on the protein of EGFR, AKT and ERK with the stimulation of mEGF or hEGF. Western blotting of p-EGFR, t-EGFR, p-AKT, p-ERK, t-AKT and t-ERK after the stimulation of mEGF or hEGF were done. β-actin was included as a loading control. The ratio of different proteins to β-actin was calculated by the band density of Western blots using Image J software. Results are presented as the median of 3 independent experiments (*P < 0.05, ^#^P < 0.01).

Next, we added mEGF to explore whether the synergistic growth inhibitory effects obtained by the combination of metformin and gefitinib, was due to a greater extent suppression of EGFR signaling via jointing at Akt and Erk. As shown in Fig. 6A–C, phosphorylation of Erk and AKT increased after mEGF stimulation. Either metformin or gefitinib treatment alone reduced these increases with further reduction while combining these two drugs together. These results demonstrated that combinations of metformin with gefitinib inhibited tumor cells growth through reducing phosphorylation of AKT and ERK synergistically.

### Effects of combination of metformin and gefitinib intravesically on syngeinic orthotopic bladder cancer mice

Orthotopic mouse model was established to provide the useful tool to determine the effect of intravesical localized treatment^21^. Syngeinic tumor implantation provided better tumor take rate compared to the xenograft implantation^22^.

**Figure 7:**
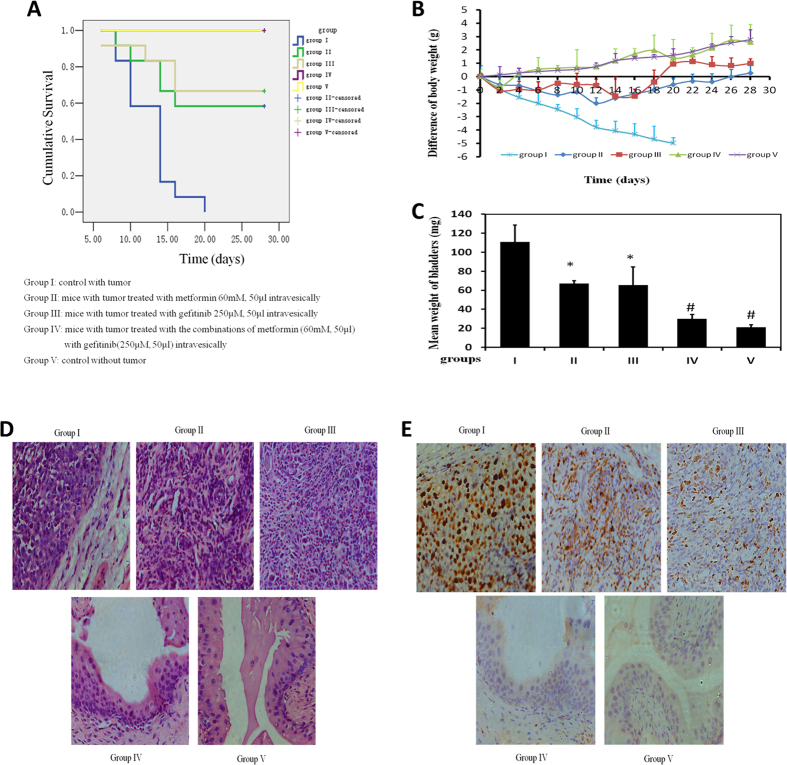
Bladders of female C57/BL6 mice were orthotopically implanted with MB49 cells (1.2 × 105) and divided into 4 groups randomly (n = 12 each group). Orthotopic bladder cancer implantation was processed for mice in groups I, II, III, and IV. Group V was mice without treatment without cancer cell implantation. Groups I, II and III were treated with 0.5% DMSO, 60 mM metformin and 250 μM gefitinib intravesically, respectively. Groups IV were intravesically treated with 50 μl both 60 mM metformin and 250 μM gefitinib, starting at day 2, twice per week for two weeks. (**A**) Kaplan-Meier survival analysis of five groups. Death of mice was checked daily and cumulative survival rate was plotted against the time course. The weight of mice was measured daily (**B**) and all living mice were sacrificed at day 28. (**C**) Weights of mouse bladders including those died before the end of experiment were measured. All bladder tissues were collected and fixed. Histological sections from these tissues were subjected to H&E stain or immunohistochemistry for Ki67 to confirm the presence or absence of tumors (**D,E**).

A total of five groups were designed: I-control with tumor and V- control without tumor, and 3 intravesical treatment groups: II- metformin-treated group (60 mM, 50 μL, equivalent to 19.5 mg/kg, twice per week), III- gefitinib treated group (250 μM, 50 μL, twice per week), and IV- combinations of metformin with gefitinib treated group (metformin 60 mM, Gefitinib 250 μM, twice per week). All treatments started at day 2 post tumor implantation for two weeks. Orthotopic bladder cancer implantation was processed for mice in Groups I, II, III, and IV except Group V, control mice without treatment in the absence of tumor. Figure 7A shows cumulative survival curves of five groups. Cancer cell implantation induces death of mice (Group I) but intravesical treatment of metformin with gefitinib dramatically enhanced life span with better survival (Group IV vs Group I, p = 0.003). es. (Group II vs Group I, p = 0.018; group III vs group I, p = 0.016). Treatment with either metformin or gefitinib alone improved mice survival respectively. As we demonstrated, decrease of mouse body weight could be a surrogate for tumor progression. Figure 7B demonstrated the decrease of body weight in Group I, indicating the toxicity induced by tumor implantation as we expected. This decrease in body weight is attenuated by intravesical treatment with metformin or gefitinib (Groups II and III) and the combination of metformin and gefitinib is able to reverse this decrease. At the end of experiment, the weight of bladders was examined. The bladder weights in Group I were much bigger than these in Group V, indicating 100% existence of tumors in all mice in Group I (Fig. 7C).

Intravesical treatment with Gefitinib or metformin alone profoundly diminished bladder weights and the bladder weights with combination of metformin and gefitinib are almost back to normal (Group IV vs Group V, p = 0.058). Furthermore, H&E results show the complete absence of tumor in Group IV (Fig. 7D) and the results of Ki67 staining demonstrates similar efficiency pattern. All together, intravesical treatment of combined drugs exhibits potent anti-cancer effect.

During our experiment, no obvious side effects such as vomiting, gastrointestinal or hematuria were observed in all drug-treated groups although we saw hematuria in group I.

## Discussion

In this study, we report pre-clinical results of intravesical treatment with metformin, gefitinib alone and the combination of these two drugs using a syngeneic orthotopic bladder cancer model.

Bladder cancer, one of the most common urinary tract cancer, frequently overexpresses EGFR on the luminal surface^23^,^24^, whereas such expression is uncommon on the normal urothelium. Therefore, it is an available choice to target EGFR for the management of bladder cancer. However, there are no targeted agents approved by FDA so far, in spite of a large scale of studies focused on EGFR has been conducted. The main obstacle is the high biodiverisity of bladder cancer and targeting a specific pathway usually causes “cross talk”, in which the neoplastic cells use as a secondary growth pathway.

Metformin, a widely used anti-diabetic drug now draws much attention since its anti-tumor activity *in vitro* and *in vivo*. The basis of molecular mechanism of anticancer of metformin is activation of AMPK, which is closely associated with tumor cells metabolism. Advances in cancer metabolism research increased the clinical interest to target aberrant metabolic pathways for treatment of malignant tumors^25^,^26^.

We combined metformin with Gefitinib, attempting to suppress bladder cancer through targeting metabolism and EGFR signaling pathway. Our *in vitro* results showed that the combinations of metformin and gefitinib induced strong inhibition on bladder cancer cells proliferation and colony formation synergistically. The apoptosis assay further confirmed the synergistic inhibitory effect on these three bladder cancer cell lines. The main mechanisms of gefitinib are the activation of AMPK signaling pathway interfered with tumor cells metabolism and inhibition on the EGFR signaling pathway significantly. Metformin, an AMPK activator, decreased the phosphorylation of AKT, ERK at the same time and showed little effect on total AKT or ERK. Although other studies observed the increased phosphorylation of ERK^27^, our results here demonstrated the decreased phosphorylation of ERK which is the most accepted concept with consistent observations^28^,^29^,^30^. This may be due to the difference of cell types.

In brief, metformin and gefitinib cooperate to inhibit bladder cancer growth via both AMPK and EGFR pathways joining at Akt and Erk and the result were further confirmed through activating EGFR signaling using mEGF.

Finally, we evaluated antitumor effect of the combination of these two drugs in syngeinic orthotopic mouse model through intravesical administration. *In vivo* experiments showed similar results to those obtained *in vitro* without evident toxicity in animals, suggesting that the combination of metformin with gefitinib may be a potential strategy for the treatment of bladder cancer.

## Material and Methods

### Reagents

Metformin (1,1-dimethylbiguanide hydrochloride) and gefitinib were purchased from Aladdin chemistry Co. Ltd and Selleck-Biotool Co. Ltd, respectively. Metformin was diluted across a range of concentrations in culture media and gefitinib was dissolved in DMSO to prepare the stock solution of 5 mM. Antibodies for the protein characteristics were against total EGFR, phosphor-EGFR, total Akt, phosphor-Akt(Ser473), total Erk1/2, phosphor-Erk1/2, phosphor-Acetyl-CoA Carboxylase (Ser79), phosphor-p70 S6 kinase(Thr389), phosphor-AMPKα (Thr172) and β actin (Cell Signaling, Beverly, MA, USA) and they were purchased from Cell Signaling Technology. Apoptosis Detection kit (FITC Annexin V) was purchased from BD Pharmingen (San Diego, California, USA).

### Cell lines and culture conditions

Murine and human bladder cancer cell lines provided by Dr. P Guo were cultured in DMEM supplemented (Hyclone, Logan, UT, USA) with 10% of FBS (Hyclone, Logan, UT, USA) and 1% of penicillin-streptomycin at 37 °C, in humidified air containing 5% of CO_2_.

### Cell Viability and cologenic Assay

Cell viability was assessed using a tetrazolium-based assay using microplate reader (Biotek, SYNERGY HTX, Vermont, USA). IC_50_ values were determined through the dose-response curves.

Cologenic survival was defined as the ability of the cells to form colonies. Images were taken and analyzed by microscopy (Leica, DFC450C, Wetzlar, Germany) and microplate reader (Biotek, SYNERGY HTX, Vermont, USA).

### Assessment of apoptosis

Apoptosis was detected by flow cytometry via the examination of altered plasma membrane phospholipid packing by lipophilic dye Annexin V. Briefly, treated cells were harvested by trypsin, washed twice with PBS, and then resuspended in binding buffer at a concentration of 1 × 10^6^ cells/mL according to the manufacturer’s instruction. Thereafter, 5 μL of Annexin V-FITC and 5 μL of propidium iodide were added into 100 μL of cell suspension and incubated for 30 minutes at room temperature in the dark. After adding 400 μL of binding buffer, labeled cells were counted by flow cytometry within 30 minutes. All early apoptotic cells (Annexin V–positive, propidium iodide–negative), necrotic/late apoptotic cells (double positive), as well as living cells (double negative) were detected by FACSC alibur flow cytometer and subsequently analyzed by Cell Quest software (Becton Dickinson). Argon laser excitation wavelength was 488 nm, whereas emission data were acquired at wavelength 530 nm (FL-1 channel) for fluorescein isothiocyanate (FITC) and 670 nm (FL-3 c3 channel) for propidium iodide.

### Protein characterization

Western blot assessment was performed using regular procedure in previous work^20^. Primary antibody was added in BSA and allowed to incubate overnight at 4°C, after washed with TBS/0.05% Tween-20 before secondary antibody was added and incubated for an additional hour at room temperature. The membrane was again washed 3 times before adding Pierce Super Signal chemiluminescent substrate (Rockford, IL, USA) and then immediately imaged on Chemi Doc (Bio-Rad, Hercules, CA, USA). The films were scanned using EPSON PERFECTION V500 PHOTO and quantified by Image J (NIH, Bethesda, MD, USA).

### Animals

Female C57BL/6 mice were purchased from Hunan SJA Laboratory Animal Co., Ltd (Changsha, Hunan, China). Animals were housed 4 per cage in a specific pathogen-free animal facility. The experimental protocol (158-001) was reviewed and approved by the Institutional Animal Care and Use Committee at Hunan Normal University. All experiments were performed in accordance with relevant guidelines and regulations.

### Orthotopic implantation and intravesical treatment

Exponential growth of MB49 cells^31^ were harvested and cell density in collection tube was counted by cell counter. Female mice 6 to 8 weeks of age were used for cancer cell implantation. The entire procedure of orthotopic implantation and intravesical treatment was similar to the previous published work^21^ except that catheter scratching is much harder to guarantee 100% tumor cell implantation rate.

### Histologic analysis

Tissue processing, H&E and Ki67 staining of 7-μm tissue sections were conducted by Department of Pathology, Hunan Provincial Cancer Hospital, Changsha, Hunan, P.R. China. The slides were reviewed by a pathologist, Lei Xue. The pathology evaluation was done to confirm the presence or absence of tumor.

### Statistical analysis

All data are presented as mean ± SEM. Statistical analyses were carried out using t test and statistical significance was assumed at a value of p < 0.05.

## Additional Information

**How to cite this article**: Peng, M. *et al*. Metformin and gefitinib cooperate to inhibit bladder cancer growth via both AMPK and EGFR pathways joining at Akt and Erk. *Sci. Rep.* **6**, 28611; doi: 10.1038/srep28611 (2016).

## Figures and Tables

**Table 1:** Inhibitory concentration 50% (IC_50_) for gefitinib.

	Bladder cancer cell lines		
Gefitinib (μM)	MB49	T24	UMUC3
	0.75	25.74	25.90
